# Sjögren’s syndrome: novel insights from proteomics and miRNA expression analysis

**DOI:** 10.3389/fimmu.2023.1183195

**Published:** 2023-05-18

**Authors:** Sarah Kamounah, Maria Lynn Sembler-Møller, Claus Henrik Nielsen, Anne Marie Lynge Pedersen

**Affiliations:** ^1^ Section for Oral Biology and Immunopathology/Oral Medicine, Department of Odontology, Faculty of Health and Medical Sciences, University of Copenhagen, Copenhagen, Denmark; ^2^ Center for Rheumatology and Spine Diseases, University Hospital Rigshospitalet, Copenhagen, Denmark

**Keywords:** Sjögren’s syndrome, proteomics, miRNA - microRNA, salivary glands, saliva

## Abstract

**Introduction:**

Sjögren’s syndrome (SS) is a systemic autoimmune disease, which affects the exocrine glands leading to glandular dysfunction and, particularly, symptoms of oral and ocular dryness. The aetiology of SS remains unclear, and the disease lacks distinctive clinical features. The current diagnostic work-up is complex, invasive and often time-consuming. Thus, there is an emerging need for identifying disease-specific and, ideally, non-invasive immunological and molecular biomarkers that can simplify the diagnostic process, allow stratification of patients, and assist in monitoring the disease course and outcome of therapeutic intervention in SS.

**Methods:**

This systematic review addresses the use of proteomics and miRNA-expression profile analyses in this regard.

**Results and discussion:**

Out of 272 papers that were identified and 108 reviewed, a total of 42 papers on proteomics and 23 papers on miRNA analyses in saliva, blood and salivary gland tissue were included in this review. Overall, the proteomic and miRNA studies revealed considerable variations with regard to candidate biomarker proteins and miRNAs, most likely due to variation in sample size, processing and analytical methods, but also reflecting the complexity of SS and patient heterogeneity. However, interesting novel knowledge has emerged and further validation is needed to confirm their potential role as biomarkers in SS.

## 1 Introduction

Sjögren’s syndrome (SS) is an autoimmune disease characterised by chronic lymphocytic infiltration of exocrine glands, particularly the salivary and lacrimal glands, leading to tissue destruction and, subsequently, glandular dysfunction. The predominant symptoms are oral and ocular dryness occurring due to hyposalivation and keratoconjunctivitis sicca, respectively, accompanied by arthralgia and fatigue (1, 2). The disease may occur alone and is then designated primary SS (pSS), or together with another autoimmune connective tissue disease, most often rheumatoid arthritis (RA), systemic lupus erythematosus (SLE) or systemic sclerosis (SSc), in which case it is designated secondary SS (sSS) (1, 2). SS can affect people of any age, but symptoms usually appear between the ages of 45 and 55. It affects ten times as many women as men (3).

The aetiology and pathogenesis of SS are not fully understood. It has been suggested that exposure to specific environmental factors (e.g. virus) in susceptible individuals results in dysregulation of the innate immune system involving the interferon (IFN) pathway (2, 4). Current evidence suggest that activated T cells are involved in the pathogenesis of SS by producing pro-inflammatory cytokines and by mediating B-cell-hyperactivity (5). The lymphocytic infiltrates observed in the salivary gland tissue mainly comprise CD4+ effector T cells, including IFN-γ-producing Th1 cells, IL-17-producing Th17 cells and IL-21-producing T follicular helper cells. Furthermore, CD4+ T-cell lymphopenia and increased number of circulating follicular helper T cells are often observed in the blood from patients with pSS (6). B-cell hyperactivity leads to autoantibody production, and in some cases, development of lymphoproliferative malignancy (2, 7). Anti-SSA/Ro and/or anti-SSB/La are found in about 70% of patients with pSS, often together with ANA (anti-nuclear antibodies) positivity. Hypergammaglobulinaemia is also prevalent as well as a positive rheumatoid factor (RF) (8). There is evidence to suggest that the salivary gland epithelial cells play a central role in regulation of the local autoimmune responses by inducing B-cell activation and survival of B cells within the target tissue (9, 10).

SS is associated with the human leukocyte antigen (HLA) class II types DR3 and DR2, particularly the DRB1*03/DQB1*02 and DRB1*15/DQB1*01 haplotypes, but only in patients having serum autoantibodies against SSA/Ro and SSB/La antigens (11–13). A recent, large study confirmed the absence of HLA association in patients with pSS negative for SSA and/SSB antibodies (14). On the basis of HLA association, presence of SSA and/or SSB autoantibodies, age of onset and clinical manifestations, it has been suggested that patients with pSS may be divided in two distinct subgroups (14).

Classification of pSS is currently based on the American College of Rheumatology-European League Against Rheumatism (ACR-EULAR) classification criteria utilizing the weighted sum of 5 objective aspects of pSS. These include anti-SSA/Ro antibody positivity, presence of focal lymphocytic sialadenitis with a focus score ≥1 in a labial salivary gland biopsy, an abnormal ocular staining score ≥5 (or van Bijsterveld score ≥4), a Schirmer’s test ≤5 mm/5 min and/or an unstimulated salivary flow rate ≤0.1 mL/min (15). None of the paraclinical findings, symptoms or disease manifestations are pathognomonic for SS, but may be observed in other autoimmune connective tissue diseases like RA and SLE (16). Furthermore, the highly variable symptomatology, autoantibody reactivity, histological features and gland functionality encompassed by the diagnosis of pSS suggest the existence of several different aetiopathogenic disease subtypes that may benefit from different treatment regimes. In order to characterize such subtypes, reliable biomarkers need to be identified. Moreover, due to the lack of distinctive clinical features and biomarkers, pSS may be diagnosed at a late stage where irreversible tissue damage has occurred. The diagnostic procedures currently in use are complicated, invasive and often time-consuming. This underlines the need for identification of new, ideally non-invasive, immunological and molecular biomarkers that can simplify the diagnostic work-up and stratification of patients and assist in monitoring the disease course and outcome of therapeutic interventions.

Recent and emerging advances within omics technologies offer opportunities to detect novel and reliable molecular diagnostic biomarkers, which can provide a higher sensitivity and specificity than the simple biomarkers and measures used today, and at the same time improve our insight into the aetiopathogenesis of SS and its subtypes. This systematic review provides an overview of current findings on proteomics and miRNA-expression profile analyses in SS, and presents the methods and biological materials used, such as saliva, blood and salivary gland tissue, for characterization of the proteomic and miRNA profiles.

## 2 Methods

**Figure 1:**
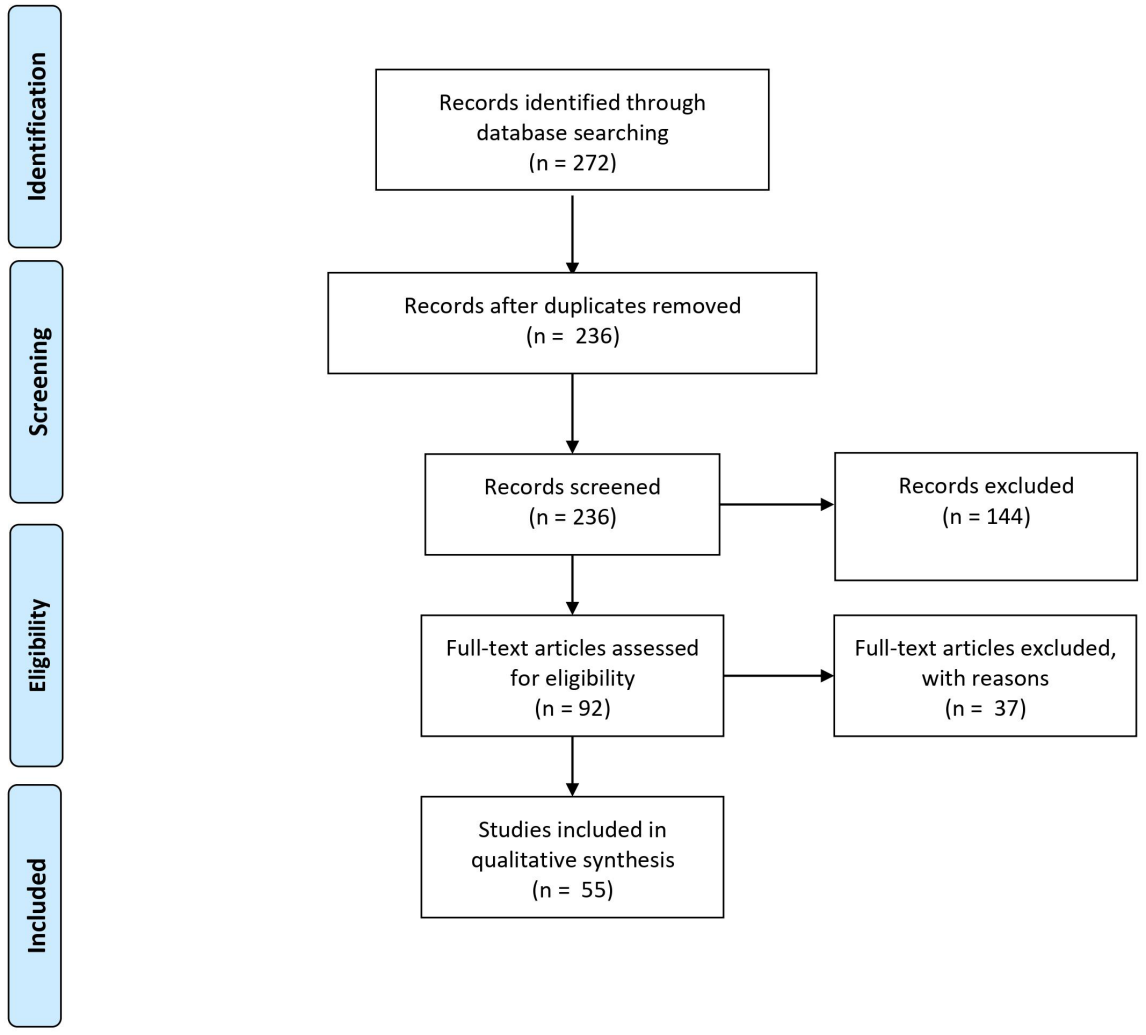
PRISMA diagram showing the number of records identified through database search, and the number of records screened and excluded.

A literature search was conducted through the PubMed, Medline and Embase databases up to January 2021 according to the standards of the *Preferred Reporting Items* for *Systematic Reviews and Meta-Analyses* (PRISMA) guidelines (**Figure 1**). Human studies that were published in English, without any restriction on publication date, and that examined proteomics and miRNAs in patients with SS (with and without comparison to healthy control subjects, or patients with other connective tissue diseases) using various methods and biological material (saliva, salivary gland tissue and blood) were included. Only primary publication types were included. Studies including less than 5 patients with SS were excluded. The following terms were used in combination to identify studies on proteomics in patients with SS: (“Sjögren’s syndrome”[MeSH]) AND “Proteomics”[MeSH]) OR Sjögren’s* proteomic* OR Sjögren’s proteomics OR primary Sjögren’s syndrome proteomic* OR primary Sjögren’s syndrome proteomics). The following terms were used in combination in the search of studies on miRNA expression-profile in patients with SS: “Sjögren’s syndrome”[MeSH]) AND “MicroRNAs” [MeSH]) OR primary Sjögren’s syndrome miRNA OR primary Sjögren’s syndrome microRNA.

Titles and abstracts were screened by two reviewers (SK and AMLP). If eligible based on the title and abstract, full-text articles were obtained when possible.

### 2.1 Saliva as a source of biomarkers

Saliva is an attractive source of biomarkers for diagnostic purposes, as salivary glands are the very organs affected by the disease, and saliva may reflect local and, possibly, systemic pathological changes of the disease (17–22). Furthermore, saliva samples can easily be obtained using a non-invasive, simple and safe procedure, and collection of saliva is thus ideal for monitoring of disease progression. Studies on the proteome in SS have mainly been performed on saliva, and stimulated whole saliva has proven more suitable for studying the salivary proteome than gland-specific saliva (23). There is also evidence suggesting that stimulated whole saliva contains a lower proteome variability than unstimulated whole saliva (24). In addition, the unstimulated whole saliva flow rate is often significantly reduced in patients with SS, which makes chewing-stimulated whole saliva more applicable. As the lacrimal glands are major target organs of SS, tear fluid represents another relevant biological material for identification of disease biomarkers. However, it may be challenging to obtain sufficient amount for analysis, and it is more complicated to collect than saliva (25).

### 2.2 Methods used for proteomic and miRNA profiling

Proteomic analysis provides insight to understand the function of proteins in health and disease. Most studies use mass spectrometry-based proteomics to obtain information on the proteome composition (e.g. shortgun gel-free proteomics) or protein interactions and structure by means of e.g. high-performance liquid chromatography-mass spectrometry (HPLC-MS), tandem mass-spectrometry (MS/MS) and matrix-assisted laser/ionization time-of-flight MS/MS (MALDI-TOF MS/MS). The methods most commonly applied for purification of proteins include absorbance colorimetry, liquid chromatography (LC), native and denaturing one-dimensional and two-dimensional gel-eletrophoresis (2-DGE), and Western blotting. LC separates proteins according to various properties and thus complements subsequent electrophoretic separation.

The method most commonly used for quantification of miRNA is real time (RT)-PCR.

## 3 Results and discussion

**Figure 1** summarizes the number of records identified through database search, and after removal, as well as the number of records screened and excluded. A total of 42 and 23 articles on proteomic profile and miRNA expression profile, respectively, were eligible for inclusion. After screening, a total of 65 full-text articles could be included for further review. These articles were published between 2006 and 2020.

### 3.1 Proteomic analysis

All studies except 4 used a cross-sectional design, including two experimental studies (26, 27), one case-control study (28) and one longitudinal study (29).

In 22 studies, proteomic analysis was performed on whole saliva (unstimulated or stimulated) or saliva collected from individual glands (19, 21–23, 25, 26, 28, 30–43).

Four studies included proteomic analysis of salivary gland tissue (22, 39, 44, 45) and 19 studies included proteomic analyses of plasma, serum or peripheral mononuclear blood cells (PMBC) (22, 27, 29, 46–63). Two studies included proteomic analysis of both stimulated whole saliva and tear fluid and of extracellular vesicles of both saliva and tear fluid from patients with pSS, healthy controls and non-pSS, sicca controls (21, 25). One study simultaneously characterized the proteome in three different types of biological materials (whole saliva, plasma, and salivary gland tissue) from the same patients and non-pSS, sicca controls (22).

Based on their aim, study design and findings, the papers on proteomics could be divided into the following categories:

1. Comparative proteomic analysis of saliva, salivary gland tissue and/or blood from patients with pSS and healthy control subjects and/or non-pSS, sicca control subjects (21–23, 25, 30, 31, 33–35, 37, 38, 40, 43, 45, 52, 54, 57, 58, 60–62)
2. Comparative proteomic analysis of saliva, salivary gland tissue and/or blood from patients with pSS and patients with RA, SLE, systemic sclerosis (SSc), mixed connective tissue disease (MCTD), polymyositis and dermatomyositis (26, 28, 36, 39, 50, 53, 59, 63).
3. Proteomic analysis of saliva and/or serum specifically related to autoantibodies in patients with SS (pSS or sSS) and control subjects (27, 29, 32, 46–49, 51, 55, 56).
4. Proteomic analysis of saliva in SS patients with and without non-Hodgkin MALT (mucosa-associated lymphoid tissue) lymphoma (41, 42, 44).

### 3.2 Comparative proteomic analysis of saliva, salivary gland tissue and/or blood from patients with pSS, healthy control subjects and/or non-pSS, sicca control subjects

#### 3.2.1 Proteomic analysis of saliva

**Table 1** presents an overview of studies on the salivary proteome of patients with pSS compared to healthy controls and patients with non-pSS sicca. The number and type of differentially expressed salivary proteins identified vary between studies. This most likely reflects the heterogeneity of SS and the underlying complexity of the inflammatory immune responses leading to varying degrees of organ damage. Overall, the studies report upregulation of inflammatory mediators and downregulation of proteins of salivary acinar cell origin in SS patients compared to non-SS patients and healthy controls.

**Table 1:** Proteomic analysis of saliva, salivary gland tissue and/or blood from patients with pSS and healthy control (HC) subjects and/or non-pSS, sicca control subjects.

Author (year)	No. of subjects	Materials and methods	Findings
Ryu OH, et al., 2006 (30)	41 pSS 15 non-pSS, sicca 5 HC	PS 2-DGE, SELDI-TOF-MS, ELISA	SELDI-TOF-MS of 10-200 kDa peaks revealed 8 peaks with >2-fold changes in the SS group that differed from non-SS (p< 0.005). Peaks of 11.8, 12.0, 14.3, 80.6 and 83.7 kDa were increased, while 17.3, 25.4, and 35.4 kDa peaks were decreased in SS samples. 2D-DIGE identified significant ↑ of β2-microglobulin, lactoferrin, immunoglobulin (Ig) kappa light chain, polymeric Ig receptor, lysozyme C and cystatin C in all stages of SS. Two presumed proline-rich proteins, amylase and carbonic anhydrase VI, were ↓ in the patient group.
Hu S, et al., 2007 (23)	10 pSS 10 HC	WS, PS, sublingual/sub-mandibular saliva MALDI-TOF-MS, 2-DGE, LC-MS/MS, RT-PCR, immunoblotting	Sixteen salivary proteins were ↓ and 25 were ↑ in pSS patients compared with HC. In pSS, the levels of 16 peptides (10 ↑ and 6 ↓) differed significantly from those of HC. WS contained more informative peptides and proteins than gland-specific saliva. Moreover, 27 mRNAs were significantly up-regulated (≥3-fold change) in saliva from pSS. In pSS, 19 of 27 overexpressed genes were interferon-inducible or related to lymphocyte filtration and antigen presentation, known to be involved in the pathogenesis of pSS.
Giusti L, et al., 2007 (31)	12 pSS 12 HC	UWS 2-DGE	The WS protein pattern differed between pSS patients and healthy controls, particularly carbonic anhydrase VI. A comparison of WS protein profile of pSS patients compared with the one obtained from HC revealed a set of differentially expressed proteins. These proteins were related to acute and chronic inflammation while some others were involved in oxidative stress injury.
Hjelmervik T, et al., 2009 (45)	6 pSS 6 non-SS controls	MSG tissue 2-DGE, LC-ESI-MS/MS	A total of 522 proteins were identified in pSS and non-SS controls, out of which 158 in pSS only, 91 in non‐SS controls only and 273 in both groups. Heat shock proteins, mucins, carbonic anhydrases, enolase, vimentin and cyclophilin B were among the proteins identified. The differences in the proteomes of MSG tissue from pSS patients and non‐SS controls were mainly related to ribosomal proteins, immunity and stress. Alpha‐defensin‐1 and calmodulin were among six proteins exclusively identified in pSS patients. Overall, proteins related to matrix, metabolism and stress were ↓, while IgG and serum albumin were ↑ in pSS.
Wei P, et al., 2013 (33)	12 pSS 13 HC	UWS MALDI-TOF-MS	The study investigated differences in low molecular weight salivary proteins (1-10 kDa) in pSS patients compared to HC. Seven m/z (mass-to-charge) (m/z 1068.1, 1196.2, 1738.4, 3375.3, 3429.3, 3449.7 and 3490.6 ratio peaks with significant differences between the 2 groups with 5 peptides being up-regulated and 2 down-regulated in the pSS patients compared to HC subjects. In the validation phase, 4 out of 5 pSS patients were diagnosed as pSS, and 4 of the 5 healthy controls were diagnosed as HC.
Gallo A, et al., 2013 (34)	82 pSS 17 non-pSS sicca 29 HC	UWS SELDI-TOF-MS, 2DE, MALDI-TOF-MS, immunohistochemistry	Gross cystic disease fluid protein-15(GCDFP-15)/prolactin-inducible protein (PIP) is a secretory acinar glycoprotein of 14 KDa. Proteomic analysis found that a putative peak of 16547 m/z, identified as the GCDFP-15/PIP protein, was among the best independent biomarkers for pSS to discriminate between patients and HC with a sensitivity of 96% and a specificity of 70%, with a global cross validated error of 29%. The intensity of GCDFP-15/PIP was significantly lower in pSS patients than in non-pSS sicca subjects and HC. GCDFP-15/PIP expression also correlated with salivary flow rate and focus score. Immunohistochemistry confirmed that GCDFP-15/PIP staining was faint in mucus acini, but no obvious difference were in serous acinar cells. RT-PCR showed that GCDFP-15/PIP mRNA was significantly lower in pSS patients than in non-pSS sicca patients and HC, supporting the hypothesis that the reduction of GCDFP-15/PIP is related to a decreased protein synthesis.
Deutsch O, et al., 2015 (35)	18 pSS 18 HC	UWS Affinity and immunodepletion, 2-DGE, LC-MS/MS	The use of depletion strategy before proteomics analysis increased identification ability by 3-fold. Overall, 79 biomarker candidates were identified. Proteins with the most pronounced fold changes were related to SS serum or tissue factors. Bioinformatics analyses of proteins with a >3-fold increase in SS patients showed calcium-binding proteins, defense-response proteins, proteins involved in apoptotic regulation, stress-response proteins and cell motion-related proteins. Preliminary validation by western blotting of profilin and CA-I indicated similar expression profile trends to those identified by quantitative MS.
Nishikawa A, et al., 2016 (52)	82 pSS (30 + 52) 30 HC	Serum High-throughput proteomic analysis, ELISA	82 proteins, identified as pSS-associated, were differentially expressed between 30 pSS patient and 30 HC (57 were ↑, 25 were ↓). Nine were identified as disease activity-associated biomarkers and among these, five proteins (CXCL13, TNF-R2, CD48, BAFF, and PD-L2) were validated as candidate biomarkers by ELISA in a separate pSS-validation cohort. CXCL13 exhibited the most significant correlation with the lymphadenopathy, glandular, and pulmonary domains of the ESSDAI. CXCL13, TNF-R2 and CD48 exhibited a positive correlation with the biological domain of the ESSDAI. TNF-R2 exhibited the most negative correlation with uptake in the submandibular gland.
Dalaleu N, et al., 2016 (37)	48 pSS	UWS 187-plex capture antibody-based assay	The study identified disease-phenotype driven biomarker signatures. Hyposalivation was associated with significant alteration in 22 out of 119 reliably detectable biomarkers. Thereof, a 4-plex signature allowed accurate prediction of salivary gland function for >80% of the cases. With respect to histopathological features, the most distinct profiles were identified in conjunction with ectopic germinal centers. Pregnancy-associated plasma protein A, thrombospondin 1 and peptide YY could recapitulate the presence or absence of tertiary lymphoid organization for 93.8% of the patients. Functional annotation of alterations associated with hyposalivation identified the IL1 system as a dominant pro-inflammatory component, while changes observed in context with ectopic lymphoid organization revealed specific shifts in chemotactic profiles and altered regulation of apoptotic processes.
Chaudhury NMA, et al., 2016 (38)	25 pSS 35 HC	UWS Western blot, LC-MS/MS	The concentration of mucins MUC5B and MUC7 were similar between patients and controls, but a comparison of protein Western blotting and glycan staining identified a ↓ in mucin glycosylation in SS, particularly on MUC7. Analysis of O-glycans released from MUC7 by β-elimination revealed an increase in core 1 sulfation, but an even larger reduction in sialylation resulting in a global decline of charged glycans. This was primarily due to the loss of the extended core 2 disialylated structure, with and without fucosylation. A reduction in the extended, fucosylated core 2 disialylated structure on MUC7, residual mucosal wetness, and whole saliva flow rate appeared to have a negative and cumulative effect on the perception of oral dryness.
Aqrawi LA, et al., 2017 (25)	27 pSS 32 HC	SWS, EV, tears LC-MS	SWS: ↑ of neutrophil gelatinase-associated lipocalin (LCN2, innate immunity), calmodulin (CALM, cell signalling) and calmodulin-5 (CALM-5, wound repair), granulins (GRN, wound repair) and epididymal secretory protein-1 (ESP1, cholesterol homeostasis within the endosome and/or lysosome) in pSS. SWS EV: ↑ adipocyte plasma membrane-associated protein (APMAP, adipocyte differentiation), guanine nucleotide-binding protein subunit alpha-13 (GNA-13, cell signalling), WD repeat-containing protein-1 (WDR1, involved in the disassembly of actin filaments), tyrosine-protein phosphatase non-receptor type substrate-1 (SIRPA, regulates NK cells and dendritic cell inhibition) and lymphocyte-specific protein 1 (LSP1, activation of innate immune system) in pSS.
Hall SC, et al., 2017 (40)	15 pSS 15 non-pSS sicca 14 HC	USW, PS iTRAQ, Lectin affinity capture-MS	The iTRAQ analyses revealed up- and down-regulation of numerous proteins that could be involved in the disease process (e.g., histones) or attempts to mitigate the ensuing damage (e.g., bactericidal/permeability increasing fold containing family (BPIF) members). An immunoblot approach confirmed the pSS-associated ↑ of β2-microglobulin (in PS) and ↓ of carbonic anhydrase VI (in WS) and BPIFB2 (in PS). Beyond the proteome, the N-glycosites of pSS and HC samples were profiled. They were enriched for glycopeptides using lectins Aleuria aurantia and wheat germ agglutinin, which recognize fucose and sialic acid/N-acetyl glucosamine, respectively. MS analyses showed that pSS is associated with increased N-glycosylation of numerous salivary glycoproteins in PS and WS.
Tasaki S, et al., 2017 (54)	30 pSS 30 HC	Whole blood transcriptomes, serum proteomes and peripheral immuno-phenotyping SOMAmer-based capture array	Identification of SS gene signatures (SGS) dysregulated in widespread omics layers, including epigenomes, mRNAs and proteins. SGS predominantly involved the interferon signature and ADAMs substrates. Besides, SGS was significantly overlapped with SS-associated genes indicated by a genome-wide association study and expression trait loci analyses. Combining the molecular signatures with immunophenotypic profiles revealed that cytotoxic CD8 T cells were associated with SGS. Activation of SGS in cytotoxic CD8 T cells isolated from patients with pSS was observed.
Bodewes ILA, et al., 2019 (57)	63 pSS (including 22 with fatigue and 23 without fatigue 20 HC	Serum SOMAscan, ELISA	pSS vs. HC: 58 proteins were ↑ and 46 were ↓. IFN-pos. and IFN-neg. pSS patients: ↑ IgG levels, higher frequency of anti-SSA and -SSB and ↓ levels of C3 complement in IFN-pos. pSS patients. 22 fatigued vs. 23 non-fatigued pSS patients: 14 serum proteins were ↑ including SNAP-25, ENO1, UCHL1 and 2 serum proteins were ↓ in fatigued. IL36a and several complement factors were upregulated in fatigued pSS patients compared to non-fatigued.
Aqrawi LA, et al., 2019 (21)	10 pSS 15 non-pSS, sicca 10 HC	SWS, tears LC-MS	SWS from pSS vs. non-pSS sicca: ↑ of *peptidyl-prolyl cis-trans isomerase FKB1A* (T-cell regulation, upregulation of NF-kappa-B signaling), *CD44 antigen* (FOXP3 expression and regulatory T-cell suppresion), *β-2 microglobulin* (B2MG, innate immunity). SWS from pSS vs. HC: ***↑*** *Secreted Ly-6/uPAR-related protein 1* ***(*** Ach-receptor activity, cell migration and proliferation), *B2MG, Clusterin* (Innate immunity, modulates NF-kappa-B activity and TNF production). SWS-EV from pSS vs. non-pSS sicca: *CD44*, *Major vault protein* (IFN-mediated signaling, MAP kinase activity, neutrophil degranulation), *Neutrophil gelatinase-associated lipocalin* (Innate immunity, tumor-associated antigen, cell adhesion). SWS-EV from pSS vs. HC: *Ficolin-1* (Innate immunity), *CD44*, *Annexin A4* (NF-kappa-B, apoptosis, IL-8 secretion).
Qiao L, et al., 2019 (58)	26 pSS with NMOSD 34 pSS without NMOSM 30 NMOSD	Serum 2-DGE, MALDI-TOF/MS, ELISA	Nine proteins were significantly differently expressed between pSS with NMOSD and pSS without NMOSD). Serum levels of clusterin and complement factor H (CFH) were further verified by ELISA. Serum clusterin was higher in NMOSD with pSS than without (298.33 ± 184.52 vs. 173.49 ± 63.03 ng/ml, p < 0.01), while the levels of CFH were lower in pSS patients with NMOSD than without.
Cecchettini A, et al., 2019 (43)	20 pSS 20 HC	UWS Nano-HPLC-SWATH-MS	PSS patients were stratified in three subgroups according to focus score in MSG tissue and unstimulated salivary flow. 203 proteins were differentially expressed in pSS patients compared to HC with evident differences in the expression of normal constituents of the human salivary proteome (i.e. prolactin-inducible protein, proline-rich proteins, and cystatins) and several mediators of inflammatory processes. 63 proteins were shared and specifically modulated in the three subsets of pSS patients converging on several inflammatory pathways. Among them S100A protein appeared of particular interest merging on IL-12 signaling and being significantly influenced by either salivary flow impairment or intensity of immune cell infiltration in the tissue.
Sembler-Møller, et al., 2020 (22)	24 pSS 16 non-pSS sicca	SWS, plasma, MSGB LC-MS	1013 proteins were detected in SWS, 219 proteins in plasma and 3166 in MSGT. SWS: 40 proteins differed significantly between the two groups, 34 of which were ↑ in the pSS group. In pSS, proteins involved in immunoinflammatory processes were ↑, whereas proteins related to salivary secretion were ↓. The combination of neutrophil elastase, calreticulin and TRIM-29 revealed the best performance in distinguishing pSS from non-pSS with an AUC of 0.97.
Huang S, et al., 2020 (60)	8 pSS 10 HC	PBMC Immobilized metal affinity chromatography (IMAC)	787 proteins were identified as differentially expressed proteins, and 175 phosphosites on 123 proteins were identified as differentially phosphorylated proteins. Using module and hub protein analyses, 16 modules for the proteins were identified, 2 clusters for the phosphoproteins and selected the top 10 hub proteins (UBA52, MAPK1, HSPA8, ACTB, ACLY, APP, ACTN1, ACTN4, VWF, and TGFB1). Finally, 22 motifs were identified using motif analysis of the phosphosites and found 17 newly identified motifs, while 6 motifs were experimentally verified for known protein kinases.
Tian Q, et al., 2020 (61)	133 pSS 108 HC	PBMC ELISA, Immunofluorescence staining, tranfection assay, and IFN-alfa treatment.	The ↑ of PARP-9 and CXCL10 as well as their co-localization was confirmed in pSS. PARP-9 levels in LSGs ↑ with ↑ focus scores in patients with pSS. PARP-9 and DTX3L were present in the infiltrating mononuclear cells from salivary glands in female NOD/LtJ mouse models. Ingenuity Pathway Analysis networks of differentially expressed proteins demonstrated that PARP-9, STAT1, and IFN-induced protein with tetratricopeptide repeats 1 (IFIT-1) participated in the IFN-related pathway. Furthermore, PARP-9 could ↑ the expression of IFIT1 and CXCL10 in B cells. Moreover, PARP-9 and CXCL10 could be induced by IFNα in B cells.
Schmidt A, et al., 2020 (62)	45 pSS, 30 controls	Plasma Hybrid immunoaffinity targeted LC-MS	The aim was to quantify plasma levels of the chemokine CXCL10, which has been associated with many immunological disorders including pSS. The hybrid approach enabled sensitive, specific, and simultaneous quantification of total, full-length (active) CXCL10 1−77 and DPP4-truncated (inactive) CXCL10 3−77 in human plasma down to the low pg/mL level, reaching ELISA sensitivities. The ratio of CXCL10 1−77 to truncated CXCL10 3−77 was ↑ in patients with pSS and provided the highest correlation with pSS disease activity.

Arrow up: upregulated. Arrow down: downregulated.

Hu et al. (23) compared different types of saliva from patients with pSS and healthy controls, including whole saliva, parotid saliva and saliva from the sublingual/submandibular glands by means of LC-MS/MS analysis. They found that whole saliva yielded more informative peptides and proteins than specific glandular saliva. On average, 53 MALDI peaks were detected in whole saliva from patients with pSS, in contrast to only 24 peaks and 26 peaks in parotid saliva and saliva from the submandibular/sublingual glands, respectively (23). Thus, stimulated whole saliva appears to be more suitable for detection of biomarkers than glandular specific secretions. In whole saliva, 25 proteins were upregulated while 16 proteins were downregulated in patients with pSS patients compared to healthy controls, all reflecting salivary gland damage and oral inflammation in pSS (23). Furthermore, 10 peptides were upregulated and six were downregulated in pSS, while 27 salivary mRNA transcripts were upregulated (≥3-fold) in pSS. Among the 27 genes that were highly overexpressed in pSS, 13 were validated by RT-quantitative PCR, substantiating upregulation of particularly IFN-inducible protein G1P2 in pSS patients. In addition, genes related to lymphocyte infiltration and antigen presentation were found overexpressed (23).

A number of studies report upregulation of β2-microglobulin in parotid saliva (23, 30, 40), unstimulated whole saliva (28, 44), stimulated whole saliva (21–23, 32) and sublingual/submandibular saliva (23) in patients with pSS versus non-pSS patients and/or healthy controls. This supports earlier findings based on different techniques, e.g. sandwich enzyme immunoassay (64). A study found that the combination of upregulated β2-microglobulin, cathepsin D and α-enolase yielded a receiver-operating characteristic (ROC) value of 0.99 in distinguishing patients with pSS from healthy subjects (65). However, this finding has not been further validated. β-2 microglobulin is a non-glycosylated protein, and constitutes the light-chain component of major histocompatibility (MHC) class I molecules. Interestingly, salivary levels of β2-microglobulin have been found associated with the extent of lymphocytic infiltration of the salivary glands in patients with pSS (40, 66) as well as with the presence of anti-SSA/Ro serum antibodies (28).

Alpha-enolase, also known as enolase 1 (ENO1), has also been found upregulated in patients with pSS compared to healthy controls and to patients with non-SS sicca in unstimulated whole saliva (28, 41). Of note, the levels of α-enolase are elevated in serum from pSS patients with fatigue compared to the levels in serum from non-fatigued pSS patients and healthy control subjects (57). Alpha-enolase is a glycolytic enzyme that is expressed in most tissues and has been identified as an autoantigen in Hashimoto encephalopathy (67) and cancer (68). It has been suggested that α-enolase is an autoantigen in pSS, as patients with pSS had higher levels of anti-citrullinated α-enolase IgG antibodies in serum than healthy controls and patients with RA (69). However, the presence of IgA and IgG antibodies against citrullinated and native α-enolase was not associated with any clinical manifestations (69).

A number of other salivary proteins have been found upregulated in pSS, including neutrophil gelatinase-associated lipocalin (NGAL/lipocalin-2) (21, 31), calgranulin A (S100A8) (35), calgranulin B (S100A9) (28), psoriasin (S100A7) (28, 35), epidermal fatty acid binding protein (E-FABP) (21, 23, 28), ACTB protein (actin) and b-Actin fragment (23, 31) as well as immunoglobulin kappa light chain (23, 28, 30, 35). Neutrophil gelatinase-associated lipocalin (NGAL), a glycoprotein belonging to the lipocalin family, is expressed in several normal tissues, where it provides protection against bacterial infection and modulates oxidative stress through anti-apoptotic effects. Levels of NGAL increase in response to inflammation, infection, intoxication, ischemia, acute kidney injury and neoplastic transformation (70). Interestingly, levels of acinar NGAL have been found elevated in minor salivary glands from patients with pSS compared to glands from non-SS patients (71). Calgranulin B (S100A9) and psoriasin (S100A7) are calcium-binding proteins involved in regulation of cellular growth. Both calgranulin-B and psoriasin have been found highly upregulated in ductal carcinoma *in situ* of the breast and in psoriasis (72). Cecchettini et al. (43) also demonstrated upregulation of S100A proteins in patients with pSS compared to healthy controls, especially in patients with a high focus score and low salivary flow rates, indicating that these proteins play a central role in the progression of glandular dysfunction. Jazaar et al. (42) found salivary S100A8/A9 associated with MALT lymphoma in pSS (see below 1.4.). Calgranulin A (S100A8) has been found upregulated in unstimulated whole saliva and parotid saliva from patients with pSS (35, 42). However, S100A8 was absent in whole saliva samples from patients with pSS in the study by Hu et al. (17). Sembler-Møller et al. (22) combined the analysis of stimulated whole saliva, plasma and salivary gland tissue. They found that salivary proteome of patients with pSS differed from that of non-pSS patients, but no significant difference in protein expression in plasma and labial salivary gland tissue was found between the groups. Several proteins were upregulated in stimulated whole saliva including neutrophil elastase, calreticulin, tripartite motif-containing protein 29 (TRIM29), clusterin and vitronectin. The combination of neutrophil elastase, calreticulin and TRIM-29 had the best performance in distinguishing pSS from non-pSS with an AUC of 0.97 (22).

Aqrawi et al. (25) identified additional upregulated proteins including calmodulin (CALM), calmodulin-like protein 5 (CALML5), granulins (GRN) and epididymal secretory protein-1 (ESP1) in whole saliva from patients with pSS. CALM and CALML5 are both calcium-binding proteins that play a role intracellular signalling and differentiation of keratinocytes, respectively. GRN are involved in wound repair and tissue remodelling, and ESP1 is involved in cholesterol homeostasis within the endosome and/or lysosome.

The most frequently identified downregulated proteins include prolactin-inducible protein (PIP) (21, 28, 34), carbon anhydrase VI (CA6) (23, 28, 30, 31, 40); salivary α-amylases (26, 28), most of the cystatins and a number of other secretory proteins (23, 28, 30, 31). PIP is a secretory protein, which is present in glycosylated and non-glycosylated forms in human saliva. Gross cystic disease fluid protein-15 (GCDFP-15)/PIP, which binds to aquaporin 5, a water channel critical to saliva formation, has been found downregulated in pSS, and the GCDFP-15/PIP expression correlated with both the salivary flow rate and focus score of minor salivary gland biopsies (34). Salivary PIP also binds to dental enamel (hydroxyapatite) and presumably participates in the formation of the enamel pellicle (73). It has been demonstrated that PIP-deficient mice (*Pip* ^−/−^ mice) develop impaired T-helper type 1 response and cell-mediated immunity (74). The role of PIP in the pathogenesis of SS requires further investigation.

CA6 is a zinc-containing metalloenzyme secreted in the salivary glands. CA6 catalyzes the conversion of salivary bicarbonate and hydrogen ions to carbon dioxide and water. The process is essential for the maintenance of salivary pH homeostasis and prevention of demineralization of the dental enamel (75). Reduced CA6 expression could imply impairment of the salivary bicarbonate buffer capacity and may explain the occurrence of increased caries activity in patients with SS.

Cystatins are cysteine protease inhibitors, which provide protecting against proteases by regulating uncontrolled proteolysis and tissue damages (76). The family of cystatins includes cystatin S, cystatin SN, cystatin C, and cystatin D. Cystatins may differ with respect to phosphorylation and/or glycosylation. Giusti et al. (31) found reduction of cystatin SN precursor in patients with pSS compared to control subjects, whereas the other cystatins were scarcely represented or totally absent. Ryu et al. (30) found an increase in salivary levels of cystatin C (about 1.3-fold) compared to the levels in saliva from controls. The diverging results may reflect variation in the levels of cystatins in the different types of saliva, i.e. whole saliva versus parotid saliva. Cystatin C levels have been reported increased in whole saliva of patients with periodontitis, and this may also contribute to explain the conflicting results. The reported decrease in salivary α-amylases (23, 28, 30, 31) may be a result of fragmentation caused by protease endogenous truncation or to the acinar cell damage.

Hall et al. (40) confirmed upregulation of β2-microglobulin in parotid saliva and downregulation of CA6 in whole saliva based on isobaric mass tagging (iTRAQ) and lectin affinity capture mass spectrometry (MS)-based approach, but also detected downregulation of parotid saliva BPIFB2 (bactericidal/permeability increasing fold containing family B2), and increased N-glycosylation of numerous salivary glycoproteins in both parotid and whole saliva from patients with pSS compared to control subjects (40).

Finally, **Table 1** includes studies that have reported upregulated or downregulated MALDI peaks (m/z) (peptides or fragments derived from proteolytic activity in parotid or whole saliva from patients with pSS compared to healthy controls) (33).

#### 3.2.2 Proteomic analysis of salivary gland tissue

In a study by Hjelmervik et al. (45), using LC-ESI-MS/MS and 2-D PAGE, six proteins were exclusively detected in labial salivary gland tissue from patients with pSS, including α-defensin-1 and calmodulin. Alpha-defensin-1 plays a central role in virus defence and upregulates the type I IFN response genes (77) and had previously been suggested as a salivary biomarker for oral inflammation in pSS (26). Calmodulin regulates inflammatory processes, and elevated levels of calmodulin-binding proteins in the salivary glands from pSS patients have also been reported (78).

#### 3.2.3 Proteomic analysis of plasma, serum or PBMC

While Sembler-Møller et al. found no difference in the protein expression profile in plasma between patients with pSS and non-pSS sicca controls, other studies on serum, plasma and PBMC found multiple proteins to be upregulated and downregulated in patients with pSS compared to controls, which may reflect different control group designs (22). Bodewes et al. (57) identified a set of proteins that could not only discriminate pSS from healthy controls, but also distinguish fatigued-pSS patients from non-fatigued pSS patients. IFN-positive pSS patients had higher levels of IgG and anti-SSA and -SSB and lower levels of C3 complement than IFN-negative pSS patients. Several serum proteins, including neuroactive synaptosomal-associated protein 25 (SNAP-25), α-enolase, ubiquitin carboxyl-terminal hydrolase isozyme L1 (UCHL1) and IL36a as well as several complement factors were upregulated in fatigued pSS patients compared to non-fatigued pSS (57).

Another study by Qiao et al. (58) found upregulation of clusterin and complement factor H in pSS patients with neuromyelitis optica spectrum disorder (NMOSD) compared to pSS patients without this disorder. These findings suggest that serum clusterin and factor H could be potential biomarkers for pSS patients with NMOSD and play a role in the pathogenesis of the disease, but further verification is needed.

Nishikawa et al. (52) identified five proteins as disease activity-associated biomarkers, including CXCL13, TNF-R2, CD48, B-cell activating factor (BAFF), and PD-L2. CXCL13 significantly correlated to the lymphadenopathy, glandular, and pulmonary domains of the EULAR Sjögren’s syndrome disease activity index (ESSDAI). CXCL13, TNF-R2 and CD48 correlated positively with the biological domains of the ESSDAI, while TNF-R2 exhibited the most negative correlation with uptake in the submandibular gland.

In a study on PBMC (60), 787 proteins were identified as differentially expressed, and 175 phosphosites on 123 proteins were identified as differentially phosphorylated proteins. The study identified 16 modules for the proteins, two clusters for the phosphoproteins and selected the top 10 hub proteins (UBA52, MAPK1, HSPA8, ACTB, ACLY, APP, ACTN1, ACTN4, VWF, and TGFB1). Finally, 22 motifs were identified using motif analysis of the phosphosites, and the authors found 17 novel motifs, while 6 motifs were experimentally verified for known protein kinases. The differentially expressed proteins enabled discrimination of patients with pSS from healthy controls and were suggested as candidate biomarkers in the diagnosis of pSS.

### 3.3 Comparative proteomic analysis of saliva, salivary gland tissue and/or blood from patients with pSS and other inflammatory connective tissue diseases

**Table 2** shows the proteomic analysis of saliva, salivary gland tissue and/or blood from patients with pSS and other inflammatory connective tissue diseases. An experimental study with peroral pilocarpine treatment on patients with pSS, sSS and healthy controls showed that the levels of almost all of the 60% salivary proteins, which was not present or present at low levels prior to treatment in pSS, reached similar levels in unstimulated whole saliva in all groups 30-60 minutes after treatment with pilocarpine. Patients with pSS also had β-defensin-2 in their saliva and higher levels of α-defensin-1 than patients with sSS and healthy controls, indicating that α-defensin-1 and β-defensin-2 may be used as biomarkers of oral inflammation in patients with pSS (26).

**Table 2:** Comparative proteomic analysis of saliva, salivary gland tissue and/or blood from patients with pSS patients with RA, SLE, systemic sclerosis, mixed connective tissue disease (MCTD) and poly- and dermatomyositis (1.2).

Author (year)	No. of subjects	Materials and methods	Findings
Peluso G, et al., 2007 (26)	9 pSS 3 RA-sSS 3 SSc-sSS 3 SLE-sSS 9 HC	UWS HPLC-ESI-MS	In 6 of the pSS patients, saliva was collected at 30 minutes, 60 minutes, and 24 hours after taking 5 mg of pilocarpine. Before pilocarpine, 60% of salivary proteins in samples from pSS patients were not identifiable or showed lower levels than those in controls. After 30–60 minutes following pilocarpine treatment, 1/3 of the less represented proteins was found in a similar percentage in pSS and controls. Almost all of the proteins that were detectable at lower levels in pSS compared to controls reached levels similar to those in controls at 30–60 minutes after pilocarpine. The parotid gland proteins had the best response to pilocarpine. PSS patients were characterized by higher α-defensin 1 levels and presence of β-defensin 2. sSS patients showed an intermediate protein profile between that of the pSS patients and HC.
Baldini C, et al., 2011 (28)	59 pSS 40 sSS 61 HC 20 non-pSS, sicca	UWS	15 differently expressed proteins were identified in pSS samples with respect to HC, non-SS sicca, SSc-sSS and RA-sSS. pSS vs. HC: ↑ S100A9, B2M, epidermal fatty acid binding protein (E-FABP), psoriasin (S100A7), immunoglobulin k light chain (IGKC) and α-enolase. ↓ AMY1A, carbonic anhydrase VI (CA6), glyceraldehydes-3-phosphate dehydrogenase (G3PDH), cystatin SN precursor protein (CST1), prolactin-inducible protein precursor (PIP), short palate, lung and nasal epithelium (SPLUNC-2). pSS vs. non-pSS sicca: ↑ PIP, SPLUNC-2 G3PDH, AMY1A, CA6. pSS vs. RA-sSS: ↑ lipocalin. ↓ rheumatoid factor D3 light chain. pSS vs. SSc-sSS: ↓ cystatin.
Li Y, et al., 2014 (50)	60 pSS 50 SLE 50 RA 51 HC	Serum MALDI-TOF-MS	100 differential M/Z peaks associated with pSS were identified (18 were ↑ in pSS). The m/z peaks at 8,133.85, 11,972.8, 2,220.81, and 4,837.66 were used to establish a diagnostic model for pSS which could distinguish pSS from non-pSS controls with a sensitivity of 77.1 % and a specificity of 85.5 %. Its efficacy was confirmed in a blinded testing set with a sensitivity and specificity of 95.5% and 88%, respectively.
Delaleu N, et al., 2015 (36)	48 pSS 12 RA 12 HC	UWS 187-plex capture antibody-based assay	Based on a 187-plex antibody-based assay, 61 and 55 proteins were differentially expressed in pSS and RA, compared to HC. All proteins were upregulated in pSS patients, except FGF-4. Based on 4-plex and 6-plex biomarker signatures, which both included IL-4, IL-5 and clusterin, achieved accurate prediction of an individual’s group membership for at least 94% of cases.
Bosello S, et al., 2016 (39)	9 pSS 7 SLE 7 RA 7 sSS/SSc 7 sSS/SLE 7 sSS/RA 7 SSc 10 HC	UWS, MSGB HPLC-ESI-MS (UWS), immunostaining (MSGB)	↑ levels of Tβ4 in pSS compared to other subgroups. Tβ10 detectable in 66,7% of pSS subjects and 42,9% of sSS/SSc patients. Tβ4 sulfoxide detectable in 44,4% of pSS patients and 42,9% of sSS/SSc patients. Tβ4 and Tβ10 not detectable in non-ss associated patients and HC. All patients had immunoreactivity for Tβ10. Tβ4 immunoreactivity was absent in pSS and sSS/RA-patients, but present in sSS/SSc and sSS/SLE-patients. In pSS, salivary Tβ expression was generally overexpressed.
Ohlsson M et al, 2021 (59)	73 pSS (AECG) 39 SLE 46 RA 82 ANCA-SV 77 HC	Serum 393-plex antibody microarray	All 393 antibodies could discriminate between IRD’s from HC with an AUC of 0.94. Based on a panel consisting of the 40 best-performing antibodies IRD could be discriminated from HC with an AUC of 0.93. The IRD could also be discriminated from each other with AUC levels ranging from 0.79 to 0.96. 77 analytes, targeted by 114 antibodies were differentially expressed in IRD compared to HC: 326 antibodies targeting 160 analytes for SV, 207 antibodies targeting 127 analytes for SS, 127 antibodies targeting 85 analytes for SLE and 114 antibodies targeting 81 analytes for RA. ↑ immunoregulatory analytes included apolipoprotein, A1, IL-6, IL-12, TNF-alfa, IL-16, osteopontin. Antibodies targeting C3, IL-4, VEGF, SPDLY-1 etc. were ↓. Among the top 25 antibodies, most of the analytes were ↑ in SLE, RA and SS (15, 21 and 25, respectively). The majority of analytes (n = 23) were ↓ in ANCA-SV.
Thiagarajan D et al, 2020 (63)	324 pSS 374 SLE 354 RA 77 MCTD 65 PAPs 118 UCTD, 331 SSc 515 HC	Serum, PBMC ELISA, LC-MS	Significantly lower IgM anti-PC but not anti-MDA was seen in MCTD compared to HC. No significant difference in levels of anti-PC nor anti-MDA was identified between disease groups, but low levels of IgM were more prevalent in MCTD, SLE, SjS, SSc and UCTD, more for anti-PC than anti-MDA.

Arrow up: upregulated. Arrow down: downregulated.

Baldini et al. (28) identified 15 differently expressed proteins in patients with pSS compared to healthy controls, non-pSS sicca, patients with RA-sSS and SSc-sSS. S100A9, β-2 microglobulin, epidermal fatty acid binding protein (E-FABP), psoriasin (S100A7), immunoglobulin k light chain (IGKC) and α-enolase were upregulated in patients with pSS compared to healthy controls, whereas AMY1A, carbonic anhydrase VI (CA6), glyceraldehydes-3-phosphate dehydrogenase (G3PDH), cystatin SN precursor protein (CST1), prolactin-inducible protein precursor (PIP), short palate, lung and nasal epithelium carcinoma associated protein 2 (SPLUNC-2) were downregulated in patients with pSS. PIP, SPLUNC-2 G3PDH, AMY1A, CA6 were upregulated in patients with pSS compared to non-pSS, sicca patients. Lipocalin was upregulated, while rheumatoid factor D5 light chain was downregulated in patients with pSS compared to patients with RA-sSS. Lastly, a comparison with patients with SSc-sSS revealed an upregulation of cystatin in patients with pSS (28).

One hundred differential m/z peaks associated with pSS were identified by Li Y al (13)., and the m/z (mass-to-charge) peaks at 8,133.85, 11,972.8, 2,220.81, and 4,837.66 were used to establish a diagnostic model for pSS. The proteomic model could distinguish pSS from non-pSS controls (SLE, RA and healthy controls) with a sensitivity of 77.1% and a specificity of 85.5%. The efficacy was confirmed by a blinded validation study.

Two studies detected differently expressed proteins using multiplexed antibody-assay in unstimulated whole saliva and serum (36, 59). Based on a 187-plex-antibody assay, 61 and 55 proteins were differently expressed in unstimulated whole saliva in pSS and RA, respectively, compared to healthy controls (36). A 4-plex and 6-plex biomarker signature was then developed, including IL-4, IL-5 and clusterin, which achieved accurate prediction of an individual group in at least 94% of the cases (36). Ohlsson et al., 2020 (59) used a 393-plex antibody microarray to test the discriminatory performance in distinguishing between different inflammatory rheumatic diseases (i.e., pSS, SLE, RA and antineutrophil cytoplasmic antibody-associated systemic vasculitis (ANCA-SV) from healthy controls in serum. An analysis including all 393 antibodies could discriminate the patient groups from the healthy controls (HC vs. SLE+RA+SS+SV) with an AUC of 0.94. Serum samples from patients with pSS could be distinguished from the other patient samples (SS vs. SLE+RA+SV) with an AUC of 0.80. A total of 207 antibodies targeting 127 analytes were found to be differentially expressed in patients with pSS compared to healthy controls.

Bosello S. et al. (39) investigated the salivary levels of Tβ4, Tβ10, and Tβ4 sulfoxide in patients with pSS, SLE, RA, SSc, sicca syndrome + SSc (ss/SSc), sicca syndrome + SLE (ss/SLE), sicca syndrome + RA (ss/RA) and healthy controls. Tβ10 and Tβ4 sulfoxide were not detectable in healthy controls and patients without sicca syndrome. Patients with pSS had higher levels of Tβ4 than the other patient groups. Tβ10 and Tβ4 sulfoxide were detectable in 66.7% and 44.4% of the patients with pSS, respectively. Overall, the patients with pSS displayed highest expression of salivary Tβ, whereas Tβ4 immunoreactivity in minor salivary glands was completely absent in patients with pSS.

### 3.4 Autoantibodies in patients with SS

Ten studies eligible for inclusion focused on proteomic analysis of serum or salivary autoantibodies in patients with SS (27, 29, 32, 46–51, 55, 56) (**Table 3**). Anti‐SSA and ‐SSB antibodies are clinically important antinuclear antibodies in patients with SS. However, these antibodies may not be present in all patients with SS, and they may also be found in other inflammatory rheumatic diseases, including SLE, polymyositis and SSc, as well as in patients with primary biliary cirrhosis (PBC) and hepatitis C viral infection (79). Anti-SSA/Ro autoantibodies are the most frequently identified antinuclear antibodies directed against Ro antigens, consisting of two different proteins, Ro60 and Ro52. The Ro 60 kDa autoantigen is an RNA-binding protein, associated with one of several human Y RNAs (small non-coding RNAs, required for DNA replication). Ro52/TRIM21 is an IFN-inducible protein, which is also induced by viral infection (80). Anti‐SSB/La also recognizes the Ro‐ribonucleoprotein. Anti-Ro52 and anti-Ro60 autoantibodies are closely linked but also display different clinical associations (81). Thus, anti-SSA/Ro52/TRIM21 antibodies show a wider spectrum of disease-associations than anti-SSA/Ro60 antibodies (82). Both Ro antigens have been found expressed in the surface of ductal epithelial salivary gland cells during apoptosis indicating that the initiation of anti-SSA/Ro60 and anti-Ro52/TRIM21 responses play a role of in the pathogenesis of SS (83). In addition, anti-SSA/Ro60- and anti-SSA/Ro52/TRIM21-specific B cells compatible with plasmablasts or plasma cells have been detected in salivary glands of patients with pSS (84). Autoantibodies may be present years before the clinical symptoms of SS, and anti-SSA and anti-SSB antibodies are therefore central biomarker targets that are useful in the diagnosis of SS (85).

**Table 3:** Proteomic analysis of saliva and/or serum specifically related to autoantibodies in patients with SS and control subjects (1.3).

Author (year)	No. of subjects	Materials and methods	Findings and conclusion
Ottosson L, et al., 2006 (27)	10 pSS	Serum MALDI-TOF-MS	Two structured parts of Ro52 were identified, corresponding to the RING-B-box and the coiled-coil regions. The two subregions were independently structured. Analysis indicated Zn^2+^-dependent stabilization against proteolysis and functional Zn^2+^-binding sites in both the RING and the B-box. Oligomerization of the coiled-coil was investigated showing weak homodimer affinity, in parity with other coiled-coil domains involved in regulatory interactions. The findings form a basis for further Ro52 functional studies on the proteome level.
Van den Bergh K, et al., 2009 (46)	93 pSS 6 sSS 147 SLE, RA, scleroderma, polymysitis, MCTD and dermatomyositis	Serum Immunofluorescence, SDS-PAGE, WB, MALDI-TOF/TOF	Recombinant rat heterogeneous nuclear ribonucleoprotein H1 (hnRNP H1) reacted with 48% of sera from pSS patients and 5.2% of 153 sera from patients with other connective tissue diseases. Five of 11 pSS patients with no anti-SSA or -SSB antibodies had anti-hnRNP H1 antibodies. hnRNP H1 was suggested as novel, potential and additional diagnostic marker in pSS.
Hu S, et al., 2011 (32)	48 pSS 47 SLE 47 HC	Whole saliva PhotoArray, ELISA	Identification of 24 potential autoantibody biomarkers that could discriminate patients with pSS from both patients with SLE and healthy subjects. Four saliva autoantibody biomarkers, anti-transglutaminase, anti-histone, anti-SSA, and anti-SSB, were further tested in independent groups of pSS and SLE patients and healthy control subjects and all were successfully validated with ELISA.
Lindop R, et al., 2011 (47)	7 pSS	Serum High-resolution Orbitrap mass spectrometry (MS), 2-DE, ELISA	Proteomic analysis revealed a Ro60-peg-specific IgG1κ-restricted monoclonal autoantibody that was present in the sera of all patients and specified by a V(H)3–23 heavy chain paired with a V(κ)3–20 light chain. The public anti–Ro 60–peg clonotype was specified further by common mutations in the heavy-chain CDR1 and light-chain complementarity-determining regions. Titers and relative affinities of clonotypic IgG did not vary over the course of the disease. The expression of a Ro 60-reactive public B cell clonotype in a subset of patients with pSS as a long-lived, class-switched circulating autoantibody implies a common breach of B cell tolerance checkpoints in these patients.
Arentz G, et al., 2012 (48)	24 pSS 14 HC SLE Polymyositis SSc	Serum 2-DGE, MS	*De novo* sequencing was used to determine the clonality and V region structures of human autoantibodies directed against Ro52 (TRIM21). Anti-Ro52 autoantibodies from patients with pSS, SLE, SSc or polymyositis were restricted to two IgG1 kappa clonotypes that migrated as a single species on isoelectric focusing; shared a common light chain (VK3-20) paired with one of two closely-related heavy chains (VH3-7, VH3-23); and were public in unrelated patients. Targeted mass spectrometry using these uniquely mutated V rion peptides as surrogates detected anti-Ro52 autoantibodies in sera with high sensitivity (87.5%) and specificity (92.9%).
Thurgood LA, et al., 2013 (49)	7 pSS, anti-SSA- and -SSB pos. 2 pSS, anti-SSA pos. and anti-SSB neg. (controls) 1 asymptomic, anti-SSA and - SSB pos. (control) 4 HC	Serum High-resolution Orbitrap mass spectrometry (MS), 2-DE, ELISA	A majority of patients with linked anti-Ro60/Ro52/La responses target an NH2-terminal epitope designated LaA, which is expressed on Ro/La ribonucleoprotein complexes and the surface membrane of apoptotic cells. Autoantibody responses comprised two heavily mutated IgG1 kappa-restricted monoclonal species that were shared (public) across unrelated patients; one clonotype was specified by an IGHV3-30 heavy chain paired with IGKV3-15 light chain, and the second by an IGHV3- 43/IGKV3-20 pairing. Shared amino acid replacement mutations were also seen within heavy and light chain complementarity-determining regions, consistent with a common breach of B cell tolerance followed by antigen-driven clonal selection.
Ohyama K, et al., 2015 (51)	14 pSS 14 SLE 11 HC 7 SSc 7 AAV 7 TA 9 MCTD 8 DM	Serum Nano-LC-MS/MS	468 distinct IC-associated antigens were identified, 62 were disease-specific antigens, and least three disease-specific antigens for each of the 7 autoimmune diseases. Coiled-coil domain-containing protein 158 and spectrin were identified as potential autoantigens important to SSc and SS pathogenesis, respectively; notable titin and spectrin autoantibodies were reportedly found in SSc and SS patients, respectively. The sensitivity of each disease-specific antigen was less than 33%. Immune complexome analysis may be generally applicable to the study of the relationship between ICs and autoimmune diseases.
Liao CC et al, 2016 (53)	18 pSS 25 RA-sSS, 18 RA 25 HC	Serum Con A affinity chromatography, 1D SDS-PAGE, in-gel digestion, and protein identification by LC-MS/M	In summary, Con A-bound ITIH3 in serum was identified as a robust diagnostic biomarker enabling discriminating RA-sSS from both HC and from pSS and RA alone. The performance of antibody isotypes against citrullinated peptides was generally better than their non-citrullinated/native counterparts, in discriminating between pSS, RA and RA-sSS and, healthy controls.
Wang JJ, et al., 2016 (55)	8 pSS 3 SLE anti-Ro60 pos. 4 HC 2 asymptomatic anti-Ro60 pos.	Serum High-resolution mass spectrometry (MS)	Monospecific anti-Ro60 Igs comprised dominant public and minor private sets of IgG1 kappa and lambda restricted heavy and light chains. Specific IgV amino acid substitutions stratified anti-Ro60 from anti-Ro60/La responses, providing a molecular fingerprint of Ro60/La determinant spreading and suggesting that different forms of Ro60 antigen drive these responses. Sequencing of linked anti-Ro52 proteomes from individual patients and comparison with their anti-Ro60 partners revealed sharing of a dominant IGHV3–23/IGKV3–20 paired clonotype but with divergent IgV mutational signatures. In summary, anti-Ro60 IgV peptide mapping provides insights into Ro/La autoantibody diversification and reveals serum-based molecular markers of humoral Ro60 autoimmunity.
Wang JJ, et al., 2018 (29)	15 RF-positive pSS 5 RF-negative pSS (controls) 30 HC 13 RF-pos + anti-Ro/LA pos. asymptomatic donor	Serum, PBMC Novo mass spectrometric sequencing, IGH repertoire sequencing	RF-specific heavy-chain third complementarity-determining region (CDR3) peptides were identified by searching RF heavy-chain peptide sequences against the corresponding IGH RNA sequence libraries. Heavy-chain CDR3 peptides were used as biomarkers to track serum RF clonotypes using quantitative multiple reaction monitoring. Serum RFs were clonally restricted and composed of shared sets of IgM heavy-chain variable region (Ig VH) 1-69, 3-15, 3-7, and 3-74 subfamilies. Cryoprecipitable RFs from patients with mixed cryoglobulinemia were distinguishable from non-precipitating RFs by a higher frequency of amino acid substitutions and identification of stereotypic heavy-chain CDR3 transcripts. Potentially pathogenic RF clonotypes were detected in serum by multiple reaction monitoring years before patients presented with mixed cryoglobulinemia. Levels of Ig VH4–34 IgM-RF decreased following immunosuppression and remission of mixed cryoglobulinemia.
Burbelo PD, et al., 2019 (56)	20 SS 20 HC 20 ICIS 11 APECED without sicca 9 APECED with sicca	Unstimulated whole saliva, serum	High percentage of autoantibody seropositivity was detected against Ro52, Ro60, and La in SS, whereas few ICIS patients were seropositive. A few APECED patients had autoantibodies to Ro52 and La, but only Ro60 autoantibodies were weakly associated with a small subset of APECED patients with sicca. Additional testing of the salivary panel failed to detect autoantibodies against any of the salivary-enriched proteins in the SS and ICIS patients. However, APECED patients selectively demonstrated seropositivity against BPI fold containing family A member 1 (BPIFA1), BPI fold containing family A member 2 (BPIFA2)/parotid salivary protein (PSP), and lactoperoxidase, 3 salivary-enriched proteins. Moreover, high levels of serum autoantibodies against BPIFA1 and BPIFA2/PSP occurred in 30% and 67% of the APECED patients with sicca, respectively, and were associated with an earlier age onset of oral dryness. These findings highlight the complexity of humoral responses in different sicca diseases and provide new insights and biomarkers for APECED-associated sicca.

Patients having circulating serum autoantibodies often present more symptoms and clinical disease manifestations than patients without presence of circulating antibodies (14). It has been shown that anti-SSA/-SSB negative patients with SS have a lower prevalence of lymphoproliferative lesions and lower risk of developing lymphoma than those who are SSA and/or SSB autoantibody positive (86). Proteomic analyses of serum from patients with pSS have revealed expression of public clonotypic autoantibodies against Ro52/TRIM21 and Ro60 antigens as well as public clonotypic autoantibodies directed against an immunodominant epitope on La (47–49). The findings of shared amino acid replacement mutations within heavy and light chain complementarity-determining regions imply a common breach of B-cell tolerance checkpoints that is followed by an antigen-driven clonal selection (48, 49). Accordingly, the findings support that the humoral immune responses against protein-RNA complexes are mediated by public sets of autoreactive B-cell clonotypes leading to systemic autoimmunity. Moreover, anti-Ro52 serum autoantibodies from patients with pSS, SLE, systemic sclerosis and polymyositis were restricted to two IgG1/kappa clonotypes, and targeted mass spectrometry detected anti-SSA/Ro52 serum autoantibodies with high sensitivity and specificity compared with conventional ELISA-technique (48). Further analysis using high-resolution mass spectrometry to sequence precipitating anti-Ro60 proteomes revealed that specific immunoglobulin variable-region peptide signatures stratified anti-Ro60 from anti-Ro60/La responses, indicating that different forms of Ro60 antigen drive the humoral Ro60 immune responses (55).

An earlier study including an immunologic analysis of the stable protein regions in sera from patients with SS showed that immunodominant epitopes predominantly are localized in the structurally stable parts of Ro52 (27). Apparently, the findings have not been substantiated by further functional studies at the proteome level.

A more recent study reported a high percentage of autoantibodies against Ro52, Ro60, and La in SS, whereas only a few patients with immune checkpoint inhibitor-induced sicca (ICIS) presented with these autoantibodies. Further testing of the salivary panel did not reveal autoantibodies against any of the salivary-enriched proteins in patients with SS and ICIS (56).

Another recent study identified antibodies against heterogeneous nuclear ribonucleoprotein H1 (hnRNP H1) in serum from about 43% patients with SS (2 primary and 5 secondary) and in about 8% patients with various autoimmune connective tissues diseases, all being ANA-positive (titer >1:160). Interestingly, 45% (5/11) of the anti-SSA/anti-SSB sero-negative patients had anti-hnRNP H1 antibodies. Thus, anti-hnRNP H1 antibody represents a potential novel diagnostic marker that can be helpful in discriminating patients with pSS from patients with SLE (potentially with sSS) (46).

Hu et al. (32) identified 24 potential salivary autoantibody biomarkers that can discriminate patients with pSS from both patients with SLE and healthy subjects. Four of them, including anti-transglutaminase, anti-histone, anti-SSA and anti-SSB antibodies, were successfully validated by means of ELISA, and on independent groups of patients with pSS, SLE, and healthy control subjects (32).

A study on molecular profiling and clonal tracking of secreted rheumatoid factors (RF) in pSS found that cryoprecipitable RF clonotypes linked to vasculitis in pSS display different molecular profiles than non-precipitating RFs. Interestingly, potentially pathogenic RF clonotypes were detected in serum by multiple reaction monitoring years before patients presented with mixed cryoglobulinemia. Furthermore, levels of Ig VH4-34 IgM-RF diminished after immunosuppression and remission of cryoglobulinemia (29).

Liao et al. (53) found serum autoantibodies against the citrullinated-inter-alpha-trypsin inhibitor heavy chain (ITIH3)^542-556^ peptide in serum in patients with pSS, RA and RA-sSS. IHIT3 is one of five chains that comprises the family of inter-alpha-trypsin inhibitors (ITI), which have the ability to bind to hyaluronic acid (HA) to interact with inflammatory cells (87). Thus, the ITIH3-HA complex is potentially involved in inflammatory diseases such as RA and pSS (88). Previous studies have reported PTM (post-translational modification)-modified proteins in patients with pSS such as citrullinated Ro60 and histone 1 (89). Furthermore, salivary antibodies against cyclic citrullinated peptide (anti-CCP), a proxy for anti-citrullinated protein antibodies (ACPA), have been identified in patients with pSS (90), suggesting that citrullinated proteins can be a utilized as diagnostic biomarkers. Liao et al. (53) found that Concanavalin A (Con A)-bound ITIH3 in serum was a robust diagnostic biomarker enabling discriminating RA-sSS from both healthy controls and from pSS and RA alone. The performance of antibodies against citrullinated peptides was generally better than antibodies against their non-citrullinated/native counterparts in discriminating between pSS, RA and RA-sSS and healthy controls. In addition, we have previously found that anti-SSA alone can differentiate pSS from non-pSS with an AUC of 0.9, while combining the presence of anti-SSA positivity with the upregulation of TRIM29 protein marker raised the AUC to 0.995 with both sensitivity and specificity between 91%-100% (91).

### 3.5 Proteomic analysis of saliva and salivary gland tissue in SS patients with and without non-Hodgkin MALT lymphoma

Patients with SS, and particularly with pSS, have an increased risk of developing lymphoproliferative malignancy. About 5-10% of the patients develop non-Hodgkin lymphoma (NHL), most commonly the mucosa-associated lymphoid tissue (MALT) B-cell lymphoma type (92–94). Risk factors include swelling of the parotid gland, vasculitis, hypergammaglobulinemia, CD4+ T lymphocytopenia, low CD4+/CD8+ T-cell ratio, low complement protein levels, cryoglobulinemia and high focus score and ectopic germinal center formation in the salivary gland tissue (92, 94). The mechanisms underlying malignant transformation of a lymphoproliferative process remain elusive.

Three studies on proteomic analysis of saliva and salivary gland tissue were eligible for inclusion in this review (41, 42, 44) (**Table 4**). Hu et al. (44) aimed at clarifying differences in gene expression and proteomic profile in parotid gland tissue from patients with pSS, patients with pSS and MALT lymphoma and non-pSS control subjects (patients with oral or oro-pharyngeal squamous cell carcinoma). Six highly associated hub genes (i.e., genes involved in proteasome degradation, apoptosis, signal peptides, complement activation, cell growth and death and integrin-mediated cell adhesion, and major histocompatibility (MHC) genes) distinguished patients with pSS from non-pSS control subjects. Eight hub genes (i.e., genes involved in translation, ribosome, protease degradation, signal peptides (MHC) class I, G13 signaling pathway, complement activation, and integrin-mediated cell adhesion) distinguished pSS patients with MALT from pSS patients. A total of 115 proteins were upregulated in patients with pSS and pSS/MALT lymphoma. Twenty-five proteins were upregulated in both pSS groups compared to non-pSS control patients, 20 proteins were upregulated in pSS compared to pSS/MALT and non-pSS control patients, and 70 proteins were upregulated in pSS/MALT compared to pSS and non-pSS control patients. The identified disease hub genes represent promising targets for therapeutic intervention, diagnosis, and prognosis (44).

**Table 4:** Proteomic analysis of saliva and salivary gland tissue in SS patients with and without non-Hodgkin MALT lymphoma.

Author (year)	No. of subjects	Materials and methods	Findings
Hu S. et al., 2009 (44)	9 pSS 6 pSS/MALT 8 non-pSS controls	Parotid gland tissue 2-DGE, Weighted gene co-expression network analysis (WGCNA)	Six highly associated hub genes (involved in proteasome degradation, apoptosis, signal peptides (MHC) class I, complement activation, cell growth and death, and integrin-mediated cell adhesion) distinguished pSS from non-pSS control subjects. Eight hub genes (involved in translation, ribosome, protease degradation, signal peptides (MHC) class I, G13 signaling pathway, complement activation, and integrin-mediated cell adhesion) distinguished pSS/MALT from pSS.
Cui L. et al., 2017 (41)	6 pSS 6 pSS/MALT 6 HC	SWS 2-DGE/MS, ELISA	**↑** α-enolase, cofilin-1, annexin A2 and Rho GDP-dissociation inhibitor 2 (RGI2) in pSS and pSS/MALT. ↑ α-enolase, cofilin-1 and RGI2 in pSS/MALT compared to pSS. The combination of the latter 3 antibodies yielded AUC 0.94 (86% sensitivity, 93% specificity in pSS vs HC) and AUC 0.86 (75% sensitivity, 94% specificity in pSS vs pSS/MALT)
Jazzar AA. et al., 2018 (42)	14 pSS/MALT (SS-M) 18 pSS high-risk of MALT (SS-HR) 19 pSS low-risk of MALT 19 pSS non-risk 14 SNOX, 18 HC	UWS parotid saliva LC-MS/MS ELISA	↑ levels of S100A8(calgranulin A) and S100A9 (calgranulin B) in parotid saliva of SS-HR and SS-M patients compared to HC. ↑ levels of S100A8/A9 in UWS in SS subgroups compared to HC and SNOX. The median concentration in SS-M were higher than in HC, SNOX and non-risk pSS patients. S100A8 and -A9 were 20-fold higher in UWS than parotid saliva.

Cui et al. (41) found α-enolase, cofilin-1, annexin A2 and Rho GDP-dissociation inhibitor 2 (RGI2) overexpressed in parotid gland tissue from patients with pSS and in patients pSS and MALT (41). Moreover, the levels of anti-α-enolase, anti-coffilin-1, and anti-RGI2 antibodies were higher in patients with pSS/MALT than in patients with pSS and healthy subjects, and significantly higher in pSS patients than in healthy subjects (41). The combination of the three antibodies yielded an AUC of 0.94 with an 86% sensitivity and 93% specificity in distinguishing patients with pSS from healthy controls, an AUC of 0.99 with a 95% sensitivity and 94% specificity in distinguishing patients with pSS and MALT lymphoma from healthy controls, and an AUC of 0.86 with a 75% sensitivity and 94% specificity in distinguishing patients with pSS and MALT from patients with pSS (41) (**Table 4**). The combination of these autoantibodies may be helpful in the diagnosis of pSS and prediction of development into MALT lymphoma.

Another study investigated the potential of S100A8 (Calgranulin A) and S100A9 (Calgranulin B) as biomarkers to discriminate patients with pSS from healthy subjects and patients with pSS from patients with pSS and MALT lymphoma (42). The levels of S100A8 and S100A9 were upregulated in parotid saliva from patients with pSS compared to healthy controls. In addition, the levels of S100A8 and S100A9 were upregulated in unstimulated whole saliva of patients with pSS, pSS/MALT compared to healthy controls and disease controls (patients with non-specified sialadenitis). Furthermore, the levels were higher in pSS patients with MALT lymphoma and pSS patients with high lymphoma risk than in pSS patients with low risk of lymphoma, healthy subjects and disease controls. Interestingly, S100A8 and S100A9 levels were 20-fold higher in unstimulated whole saliva than in parotid saliva. These findings suggest that S100A8 and S100A9 can assist in distinguishing pSS patients from healthy subjects and pSS patients with MALT lymphoma from pSS without lymphoma (42) (**Table 4**). S100A8 and S100A9 are proinflammatory proteins that play a central role in the acute and chronic inflammation. They have been found associated with autoimmune diseases and upregulated in various human cancers (117, 118).

### 3.6 MiRNA analysis of blood, PBMC, saliva and salivary gland tissue

MicroRNAs (miRNAs) are short non-coding RNA molecules, which main function is to regulate gene expression posttranscriptionally by messenger (mRNA) degradation or translational repression (119). Previous findings indicate aberrant miRNA expression in PBMC (96, 98, 99, 102, 104, 109, 120) and salivary gland tissue of patients with pSS (95, 97, 121). However, the number of publications within the field of miRNA expression profiling is increasing, revealing novel biomarkers of interest.

All studies except one used a cross-sectional design, which was a longitudinal study (108) (**Table 5**). In 16 studies, the miRNA analysis was performed on plasma, serum or peripheral mononuclear blood cells (PMBC). Six studies included miRNA analysis on salivary gland tissue. We have previously performed combined miRNA analyses on whole saliva, plasma and minor salivary gland tissue (114), and Gourzi et al. (101) combined miRNA analyses on PBMC, minor salivary gland tissue and salivary gland epithelial cells.

**Table 5:** miRNA analysis of plasma, serum, PBMC, saliva and salivary gland tissue.

Author (year)	No. of subjects	Material and methods	Findings
Alevizos I, et al., 2011 (95)	16 pSS 5 HC 1 non-pSS, sicca 1 myositis 1 peripheral neuropathy	MSGT RT-qPCR	MiRNA expression patterns in minor salivary gland tissue accurately distinguished pSS patients from healthy controls. Validation of miR-768-3p and miR-574 in an independent cohort revealed increased expression of miR-768-3p, and decreased expression of miR-574 with an increasing focus score. Comparison of miRNAs from patients with preserved or low salivary flow identified a set of differentially expressed miRNA, most of which were increased in in the group with decreased salivary gland function, suggesting that the targets of miRNA may have a protective effect on epithelial cells.
Zilahi E, et al., 2012 (96)	21 pSS 10 HC	PBMC qRT-PCR	Increased expression of miR-146a and miR-146b and the gene of TRAF6 in patients with pSS compared to healthy controls. Decreased expression of IRAK1 gene suggests transcriptional repression of IRAK1 in PBMC of pSS patients, whereas the other NF-κB pathway-regulating gene, TRAF6, is overexpressed.
Tandon M, et al., 2012 (97)	6 pSS 3 HC	MSGT qPCR	Six previously unidentified miRNA sequences (hsa-miR-4524b-3p, hsa-miR-4524b-5p, hsa-miR-5571-3p, hsa-miR-5571-5p, hsamiR-5100, and hsa-miR-5572) found in patient samples and in several cell lines. Validation of hsa-miR-5100 showed decreased expression in pSS patients with low salivary flow rates.
Peng L, et al., 2014 (98)	33 pSS 10 HC	PBMC Microassay, qRT-PCR	202 miRNA were upregulated and 180 were downregulated in pSS patients compared to healthy controls. MiR-181a differed most profoundly. No difference in miRNA-181a expression between patients with different disease phenotypes.
Shi H, et al., 2014 (99)	27 pSS 22 HC	PBMC RT-PCR	Higher expression levels of miR-146a in patients with pSS than in healthy controls, and levels correlated with the VAS scores for parotid swelling and dry eyes). Low miR-155 expression level in the patients with pSS, levels correlated with the VAS score for dry eyes.
Chen JQ, et al., 2015 (100)	23 pSS 10 HC	PBMC RT-PCR	MiR-155 is regarded as a central modulator of T-cell responses. This study evaluated the expression rate of miR-155 and its functional linked gene (SOCS1). MiR-155 and SOCS1 gene was both overexpressed in the PBMC of patients with pSS. However, there was no significant correlation between expression values of SOCS1 and miR-155 in the pSS patients.
Gourzi VC, et al., 2015 (101)	29 pSS 24 non-pSS, sicca	PBMC, MSG tissue, SGEC RT-PCR	Higher levels of miR16 in minor salivary gland tissue of miR200b-3p in salivary gland epithelial cells and miR483-5p in PBMCs from pSS patients than in non-pSS, sicca controls. Levels of let7b, miR16, miR181a, miR223 and miR483-5p correlated with Ro52/TRIM21-mRNA. MiR181a and miR200b-3p correlated negatively with Ro52/TRIM21 and Ro60/TROVE2 mRNAs in salivary epithelial cells, respectively, whereas let7b, miR200b-5p and miR223 correlated with La/SSB-mRNA. In PBMCs, let7b, miR16, miR181a and miR483-5p correlated with Ro52/TRIM21, whereas let7b, miR16 and miR181a correlated with La/SSB-mRNA expression. Lower levels of miR200b-5p in pSS patients with MALT-lymphoma than in those without.
Willams AEG, et al., 2016 (102)	21 pSS 9 sSS 17 SLE 18 RA 17 HC	PBMC CD4+ qRT-PCR	Higher levels of miRNAs monocytes from patients with SS than in healthy controls. MiR-34b-3p was differentially expressed between SS patients and healthy controls and RA. Higher levels of miR4701-5p and miR-3162.3p in SS patients. QRT-PCR supported co-regulation of miR-34b-3p, miR-4701-5p, miR-609, miR-300, miR-3162-3p, and miR-877-3p in SS monocytes (43%) in comparison with SLE (5.8%) and RA (5.6%). MiRNA-target pathway predictions identified SS-associated miRNAs appear to preferentially target the TGFβ signaling pathway as opposed to the IL-12 and Toll-like receptor/NFkB-pathways.
Yan T, et al., 2017 (103)	57 pSS - group 1: FS=1 - group 2: FS=2 - group 3: FS=3 13 HC	MSGT qRT-PCR	The differences between HC and the 3 pSS subgroups were statistically significant for positive findings of salivary flow rate, Schirmer test and laboratory indices. In LMSG tissues: expression level of miR-18a was **↑** in patients of the 3 pSS subgroups compared to HC, while expression level of miR-92a was **↓**. MiR-18a was progressively **↑** along the advanced histological stages of the 3 pSS subgroups, while the miR-92a was progressively **↓**. There was no notable difference in the expression levels of miR-17, miR-19a, miR-19b, and miR-20a.
Chen JQ, et al., 2017 (104)	8 pSS 8 SLE 7 HC	Peripheral blood Illumina next-generation sequencing	135 miRNA and 26 miRNAs showed altered expression in SLE and pSS, respectively, compared to HC. The 25 miRNAs including miR-146a, miR-16 and miR-21, which were over-expressed in pSS patients, were also found to be elevated in SLE group. miR-150-5p was **↓** in pSS. Levels of several miRNAs over-expressed in SLE, were not changed in pSS, such as miR-148a-3p, miR-152, miR-155, miR-223, miR-224, miR-326 and miR-342. Expression levels of miR-223-5p, miR-150-5p, miR-155-5p and miR-342-3p, which miRNAs are potentially linked to B cell functions, showed associations with the B cell proportions within PBMC.
Jiang Z, et al., 2017 (105)	60 pSS 53 RA 23 HC 45 SSc 49 DM 11 PM	PBMC qRT-PCR	The miR-200c level in the SSc group was significantly **↑** than in the DM/PM, pSS, and RA groups, and the levels in the DM/PM and pSS groups were significantly **↑ t**han in the RA group. The level of miR-200c in the CTD+ILD group was significantly **↑** than in the CTD–ILD group, and the level in the severe ILD group was significantly **↑** than in the mild ILD group. FVC and FEV1 were significantly different among the different CTD groups, and among the different CTD+ILD groups. There was a negative correlation between the level of miR-200c and FVC and FEV1.
Lopes AP, et al., 2018 (106)	37 pSS 17 HC 21 non-pSS, sicca	Serum RT-qPCR	10 sncRNAs were differentially expressed between the groups in the array. In the validation cohort, the **↑** expression of U6-snRNA and miR-661 in the iSS group as compared to HC was confirmed, but none of the differentially expressed sncRNA from the discovery cohort were validated in the validation cohort. However, within this group several miRNAs correlated with laboratory parameters. Unsupervised clustering distinguished three clusters of pSS patients. Patients in one cluster showed **↑** serum IgG, prevalence of anti-SSB, IFN-score, and **↓** leukocyte counts compared to the two other clusters. Patients with pSS, being anti-SSA/-SBB positive, showed **↓** expression of several sncRNAs when compared to antibody-negative patients with pSS.
Kapsogeorgou EK, et al., 2018 (107)	79 pSS - 27 low-risk who did not develop lymphoma during follow-up. - 17 high risk diagnosed with NHL during follow up, 35 SS-associated lymphoma. 8 non-pSS sialadenitis - 4 associated with sarcoidosis - 4 associated with HCV infection.	MSGT qPCR	The MSG levels of miR200b-5p were significantly **↓**in pSS patients, who will develop or have NHL, and strongly discriminated (p<0.0001) them from those without lymphoma or non-SS sialadenitis. Furthermore, they were reduced long before clinical onset of lymphoma, did not significantly change on transition to lymphoma and, importantly, were proved strong independent predictors of patients, who will develop NHL (p<0.0001). MiR200b-5p levels correlated negatively with ESSDAI and biopsy focus score, and positively with serum C4 levels.
Jiang CR, et al., 2018 (108)	70 pSS 60 HC	PBMC qRT-PCR	The expressions of miR-146a and miR-4484 in the pSS group were significantly **↑** compared to HC. After treatment, the expressions of miR-146a and miR-4484 were significantly **↓** compared with those before treatment (p<0.05). Combined detection of miR-146a and miR-4484 was superior to single index detection in the diagnosis and prognosis of pSS (p<0.05). The 3-years follow-up showed that the incidences of renal injury and pulmonary interstitial lesion in patients with low miR-146a and miR-4484 expressions were significantly lower than those with high expressions (p<0.05). No significant differences in the survival rate between the two groups (p>0.05).
Wang-Renault SF, et al., 2018 (109)	Discovery cohort: 17 pSS, 15 HC Replication cohort: 27 pSS 12 HC	Purified B- and T lymphocytes RT-qPCR	In CD4+ T-cells, hsa-let-7d-3p, hsa-miR-155-5 p, hsa-miR-222-3 p, hsa-miR-30c-5p, hsa-miR-146a-5p, hsa-miR-378a-3p and hsa-miR-28-5 p were significantly differentially expressed in both cohorts. In B cells, hsa-miR-378a-3p, hsa-miR-222-3 p, hsa-miR-26a-5p, hsa-miR-30b-5p and hsa-miR-19b-3p were significantly differentially expressed. Potential target mRNAs were enriched in disease relevant pathways. There was an inverse correlation between expression of BAFF and hsa-miR-30b-5p in B cells from pSS patients. Functional experiments showed increased expression of BAFF after inhibiting hsa-miR-30b-5p.
Wang J, et al., 2019 (110)	20 pSS 20 HC	PBMC qRT-PCR, Western Blot, ELISA	Expression of miR-let-7d-3p was dramatically regulated in CD4+ T cells from pSS-ptt. Expression of miR-let-7d-3p was negatively correlated with the expression of IL-17 in pSS patients on both mRNA and protein levels. Besides, the AKT1/mTOR signaling pathway was found critical for miR-let-7d-3p-mediated IL-17 expression. AKT1 was proved to be the direct target of miR-let-7d-3p; miR-let-7d-3p targeted AKT1 to bridge the regulation of IL-17. It was also verified that AKT1 co-expression could rescue IL-17 downregulation caused by miR-let-7d-3p.
Hillen MR, et al., 2019 (111)	30 pSS 16 HC	Peripheral blood OpenArray Q-PCR-based technique	20 miRNAs were differentially expressed at a lower level in pSS pDCs compared with HC pDCs. Differential expression of 10 miRNAs was confirmed in the replication cohort (miR-29a and miR-29c showed the most robust fold-change difference between pSS and HC). The dysregulated miRNAs were involved in phosphoinositide 3-kinase-Ak strain transforming and mammalian target of rapamycin signaling, as well as regulation of cell death. In addition, a set of novel protein targets of miR-29a and miR-29c were identified, including five targets that were regulated by both miRs (FSTL1, CASP7, CD276, SEPINH1, F11R).
Gallo A, et al., 2019 (112)	5 pSS (high salivary flow) 6 pSS (low salivary flow) 5 non-pSS, sicca	MSGT In silico analysis, Human glycosylation-RT2 Profiler PCR array.	126 out of 754 miRNA were significantly deregulated in pSS vs. HC, with a trend that was inversely proportional with the impairment of salivary flow rates. In silico approach pinpointed several upregulated miRNA in patients with pSS target important genes in the mucin O-glycosylation. This was confirmed by RT-qPCR highlighting downregulation of some glycosyltransferase and glycosidase genes in pSS samples compared to HC, such as GALNT1, responsible for mucin-7 glycosylation.
Talotta R, et al., 2019 (113)	28 pSS 23 HC	UWS, plasma miRNA RT-qPCR	No significant difference in salivary miRNA expression between patients and HC. Patients with SS had higher expression of salivary miR146a than HC. Salivary miR146b was significantly more expressed in the ptt. with worse ESSPRI scores (p=0.02), whereas salivary miR17 and 146b and plasma miR17 expression was **↓** in the patients with higher ultrasound scores (respectively p=0.01, p=0.01 and p=0.04). Salivary miR18a expression was significantly **↑** in the patients who were anti-La/SSB positive (p=0.04). Neither salivary nor plasma miRNAs correlated with disease duration or concomitant therapies.
Sembler-Møller M, et al., 2020 (114)	24 pSS 16 non-pSS, sicca	WS, plasma, MSGT miRNA RT-qPCR	In saliva 14 miRNAs were significantly differentially expressed between pSS and non-pSS; 11 of these were downregulated incl. the miR-17 family in pSS. In MSGT of pSS-ptt. miR-29a-3p was significantly upregulated. Plasma miRNAs did not differ between the two groups. The combination of miR-17-5p and let-7i-5p in saliva yielded an AUC of 97% (CI 92%-100%). Several miRNAs correlated significantly with one another and with salivary flow rates and histopathology.
Yang Y, et al., 2020 (115)	8 pSS X? HC	MSGT RT-qPCR, western blot, Annexin-V-FITC, TUNEL	TRIM21-targeting miRNAs were identified, miR-1207-5p and miR-4695-3p. Transfection of miR-1207-5p or miR-4695-3p mimics lower expression of TRIM21 and the levels of pro-apoptotic genes BAX, CASP-9 and CASP-8, leading to antiapoptotic phenotypes in HSG cells. Consistent with the antiapoptotic activity, transfection of microRNA inhibitors **↑** the expression of TRIM21 and led to a pro-apoptotic phenotype. Thus, proposing that miR-1207-5p and miR-4695-3p are antiapoptotic microRNAs functioning through apoptosis pathway. Assays performed with MSGT revealed **↓** of miR-1207-5p and miR-4695-3p + **↑** of TRIM21 and pro-apoptotic CASP-8 gene in pSS patients.
Gong B, et al., 2021 (116)	13 pSS 13 HC	PBMC RT-qPCR	The numbers of aberrant miRNAs in pSS naïve (vs. healthy naïve), pSS activation (vs. pSS naïve), MSC treatment and pre−IFN−γ MSC treatment (vs. pSS activation) groups were 42, 55, 27 and 32, respectively. Gene enrichment analysis revealed 259 pathways associated with CD4+ T cell stimulation, and 240 pathways associated with MSC treatment. Increased miRNA−7150 and miRNA−5096 and **↓** miRNA−125b−5p and miRNA−22−3p levels in activated CD4+ T cells from patients with pSS were reversed by MSC treatment. Notably, the proliferation of CD4+ T cells and CD4+ IFN−γ+ cells, expression levels of miRNA−125b−5p and miRNA−155 in CD4+ T cells and supernatant IFN−γ secretion were associated with disease activity. MiRNA may play a vital role in MSC treatment for activated CD4+ T cells.

Arrow up: upregulated. Arrow down: downregulated.

As with the proteomics studies, the findings in the 23 studies were very heterogeneous in terms of number and type of differentially expressed miRNAs. Nevertheless, several studies identified similar miRNAs to be differentially expressed such as miR-146, miR-155 and miR-188.

A study by Zilahi et al. (96) found both miR-146a and miR-146b to be significantly overexpressed in patients with pSS compared to healthy controls inPBMC. Two additional studies (99, 108) supported these findings displaying a higher expression of miRNA-146a in patients with than in healthy controls in PBMC. Wang-Reanult et al. (109) found miR-146a to be differentially expressed in CD4+ T cells. Moreover, miR-146a has been found upregulated in saliva in patients with pSS and in addition, miR-146b levels correlated with ESSPRI scores, while being downregulated in patients with high salivary gland ultrasound scores (113).

MiR-155 is a central modulator of T-cell responses, and SOCS1 is its functional linked gene (100). Shi H et al. (99) reported a lower expression of miR-155 in patients with pSS. Furthermore, miR-155 correlated with VAS score for dry eyes. On the other hand, Chen et al. (100) reported higher expression of both miR-155 and SOCS1 gene in PBMC in patients with pSS. In another study, miR-155 was higher expressed in patients with SLE, but unchanged in patients with pSS (104). In a recent study, the expression levels of miR-155 and miR-125b-5p in CD4+ T cells were found associated with disease activity (116). These findings may provide insight in the pathogenesis and disease course of SS and suggest a novel target for treatment.

We identified a downregulation of several miR-17 family members (i.e. miR-17-5p, miR-106a, miR-106b, and miR-20b-5p) in patients with pSS compared to non-pSS subjects (114), which confirms the results of previous studies (109, 119, 121). The miR-17-family takes part in the miR-17-92 cluster, previously shown to be related to carcinogenesis and autoimmunity (122). In addition, we previously found upregulation of let-7i-5p in patients with pSS that was inversely correlated with salivary flow rates and positively correlated with higher focus score, suggesting that let-7i-5p canbe used as a marker for disease activity/advanced disease state (114).

Gourzi et al. (101) determined the levels of miR-181a among other miRNAs in minor salivary gland tissue and epithelial cells and PBMC in patients with pSS and non-pSS, sicca. The levels of miR-181a, let7b, miR-16 and miR-483-5p in minor salivary gland tissue correlated with Ro52/TRIM21-mRNA. On the contrary, riR181a and miR-200b-3p correlated negatively with Ro52/TRIM21 and Ro60/TROVE2-mRNA in salivary gland epithelial cells, respectively. Let7b, miR-200b-5p and miR-233 correlated with La/SSB-mRNA. In PMBCs, miR181a, miR-16 and let7b correlated with both Ro/52-TRIM21- and La/SSB-mRNA expression (101). It has previously been shown that miR-200b-5p correlate inversely with ESSDAI and focus score in labial salivary gland biopsies, and positively with serum C4 levels (107). Furthermore, Kaspsogeorgou et al. (107) found lower expression of miR-200b-5p in pSS patients with NHL or with a high risk of developing NHL compared to patients with pSS without lymphoma and patients with non-pSS sialadenitis. In fact, the levels of miR200b-5p were lower long before clinical onset of lymphoma and were proved to be strong independent predictor of patients who will develop Non-Hodgkin’s lymphoma (NHL). These findings suggest that levels of miR-200b-5p in minor salivary gland tissue may represent a predictive biomarker for development of SS-associated NHL.

Lopes et al. (106) investigated serum levels of 758 small non-coding RNAs (snRNA) including miRNAs in patients with pSS, non-pSS sicca and healthy controls. Three and nine sncRNAs were differentially expressed in the pSS-group and non-pSS sicca-group, respectively, compared to healthy controls. Two of the sncRNAs, including, U6-snRNA and miR-29c-3p, were differently expressed in both patient groups. Interestingly, the abundance of several of the differentially expressed snRNAs correlated with laboratory parameters and disease activity (i.e., low C3/C4, decreased leukocyte count, high focus score and anti-SSA/Ro and/or anti-SSB/La).

Furthermore, the specific pattern of snRNA expression could identify different disease phenotypes within the pSS group. Hierarchical clustering was performed to subgroup the pSS patients into 3 clusters based on their expression of the nine sncRNAs. One group (cluster 3) presented with an increased disease activity, including increased IgG levels, autoantibody positivity and IFN-score in their serum, decreased leukocyte count and furthermore, an overall decreased in the expression of all nine sncRNAs compared to the two other groups. It was concluded that no snRNAs could discriminate pSS from non-pSS sicca. However, the snRNA levels may reflect the disease activity and can be used to identify pSS patients with increased B cell hyperactivity. Hillen et al. (111) supported the findings of differentially expressed miR-29c as they reported miR-29a and miR-29c to be significantly downregulated in plasmacytoid dentritic cells (pDC) from patients with pSS compared to healthy controls.

Gallo et al. (112) found a higher expression of miR-18b, miR-20a, miR-106 and miR-146b in minor salivary gland tissue from patients with pSS than in those of healthy controls. These miRNAs correlated inversely with salivary flow rates. MiR-635 and miR-372 were downregulated and correlated positively with salivary flow rates. The most significant pathways that could be targeted by the 100 most regulated miRNA (>2 fold change) in minor salivary gland biopsies from the patients with pSS included the KEGG and mucin type O-glycan biosynthesis pathways. It is well-known that mucins are important for the rheological properties in saliva (123, 124). Interestingly, a glycosylation profiling analysis on salivary samples from pSS and healthy controls revealed deregulation of some glycosyltransferases and glycosidases suggesting that an alteration of miRNA-target genes in the mucin type O-glycan biosynthesis may influence the glycosylation of salivary mucins and salivary functions in pSS (112).

Two TRIM21-targeting miRNAs, miR-1207-5p and miR-4695-3p, have previously been identified (115), and their role in the development of pSS has been investigated using human submandibular gland cells. Transfection of miRNA mimics into human submandibular gland cells revealed downregulation of mRNA to 53% and 42% for miR-1207-5p and miR-4695-3p, respectively. The expression of TRIM21 was also downregulated to 18% and 33% for miR-1207-5p and miR-4695-3p, respectively. Additionally, upregulation of TRIM21 was seen after transfection of miRNA inhibitor. Findings indicate that miR-1207-5p and miR-4695-3p are anti-apoptotic in human submandibular glands cells and regulate the expression of TRIM21 and multiple pro-apoptotic genes in salivary glands (115). In pSS, an upregulated expression of TRIM21 due to decreased miR-1207-5p and miR-4695-3p may lead to upregulation of the CASP-8 and ultimately facilitates the minor salivary glands cells to undergo apoptosis (115).

Peng et al. (98) found 180 miRNAs upregulated and 202 miRNAs were downregulated in PBMC from patients with pSS. Eleven of these (miRNA-let-7b, miRNA-142-3p, MiRNA-142-5p, miRNA-146a, miRNA-148b, miRNA-155, miRNA-18b, miRNA-181a, miRNA-223, miRNA-23a and miRNA-574-3p) are involved immune regulatory mechanisms. MiR-181a was the most significant differentially expressed miRNA in patients with pSS compared to healthy controls. MiRNA-181a only correlated with ANA, suggesting that PBMC miRNA-181a is a potential biomarker to distinguish pSS from healthy controls, but cannot be used to discriminate between different disease phenotypes.

## 4 Conclusion

Overall, the proteomic and miRNA studies revealed considerable variations with regard to candidate biomarker proteins and miRNAs, most likely due to variation in collection method, sample size, processing and analytical methods, but also reflecting the complexity of SS and patient heterogeneity. Furthermore, the high rate of salivary posttranslational modifications such as glycosylation, phosphorylation, sulfation, transglutaminase and proteolytic cleavages adds structural and functional diversity to the proteome, which may challenge interpretation of the data. It should also be noted that while serum-derived proteins in saliva are mainly involved in cell cycle, signal transduction etc., salivary proteins and peptides are involved in antibacterial activity, lubrication, digestion, oral homeostasis and more likely express local changes and thus better reflect changes/diseases in the oral cavity.

Nevertheless, interesting novel knowledge has emerged and several studies support the findings of differential expression of certain proteins such as upregulation of cystatin A, β2M, α-enolase, actin, E-FABP, N-GAL, Ig-κ light-chain and C3 as well as downregulation of PRPs, CA6, α-amylase, histatins, PIP, cystatin S and cystatin SN. Another novel finding was the discriminatory performance of an anti-SSA/Ro combined with TRIM29, which showed a higher sensitivity than anti-SSA/Ro positivity alone. However, further validation with larger multicenter studies is needed to confirm their potential role as biomarkers in SS.

## Data availability statement

The original contributions presented in the study are included in the article/supplementary material. Further inquiries can be directed to the corresponding author.

## Author contributions

SK and AP: Contributed with conception of the work, data acquisition, prepared the figures and tables, and drafted the manuscript. SK, AP, MS-M, and CN critically reviewed and edited the final version of the manuscript. All authors contributed to the article and approved the submitted version.
